# Phlebotomine sand fly (Diptera: Phlebotominae) diversity in the foci of cutaneous leishmaniasis in the Surxondaryo Region of Uzbekistan: 50 years on

**DOI:** 10.1007/s00436-024-08191-4

**Published:** 2024-03-25

**Authors:** Gofur X. Usarov, Vladimir S. Turitsin, Xulkar G. Sattarova, Jovana Sádlová, Javokhir Abdusamat ugli Mustanov, Andreu Saura, Vyacheslav Yurchenko

**Affiliations:** 1 Isayev Research Institute of Microbiology, Virology, Infectious and Parasitic Diseases, Samarkand State Medical University, 140100 Samarkand, Uzbekistan; 2 St. Petersburg State Agrarian University, St. Petersburg, 196605 Russia; 3 Department of Parasitology, Faculty of Science, Charles University, 128 00, Prague, Czechia; 4 Termiz Branch of Tashkent Medical Academy, 132000 Termiz, Uzbekistan; 5 Life Science Research Centre, Faculty of Science, University of Ostrava, 710 00 Ostrava, Czechia

**Keywords:** Phlebotomus, Diversity, Leishmania

## Abstract

In Uzbekistan, the number of reported leishmaniasis cases is rising at the alarming rate. In this work, we studied the phlebotomine sand fly (Diptera: Phlebotominae) diversity in the foci of cutaneous leishmaniasis in the Surxondaryo Region of Uzbekistan and compared it with the data obtained for the same area 50 years ago, when infection prevalence was reportedly low. We found that the implicated vector for zoonotic leishmaniasis, *P. papatasi*, remained eudominant; the proportion of implicated anthroponotic leishmaniasis vector, *P. sergenti*, rose significantly from averaged 5.4 to 41.4%; *Phlebotomus alexandri*, a suspected visceral leishmaniasis vector, was eudominant at two sites, and a second suspected vector for this disease, *P. longiductus*, was newly recorded in the region. We conclude that the increase in the documented cases of cutaneous leishmaniasis in the Surxondaryo Region of Uzbekistan may be connected to the changes in fauna of sand flies vectoring *Leishmania* spp.

**Supplementary Information:**

The online version contains supplementary material available at 10.1007/s00436-024-08191-4.

## Introduction

In Uzbekistan, leishmaniasis—a neglected vector-borne disease caused by *Leishmania* spp. (Kinetoplastea: Trypanosomatidae) (Bruschi and Gradoni 2018; Kostygov et al. 2024)—is a serious problem and the number of cases is on the rise (Mustanov and Nematov 2019; Strelkova et al. 2015; Suvonkulov et al. 2020; Yurchenko et al. 2023). These parasites are dispersed worldwide and affect up to 15 million people in about 100 (mainly, tropical and subtropical) countries (WHO 2023). By clinical manifestation, leishmaniasis can be cutaneous (CL) and visceral (VL) resulting in often self-healing skin ulcers and multiorgan damage, respectively (Burza et al. 2018; Mann et al. 2021). Both forms are present in Uzbekistan (Alam et al. 2009; Mustafaev 1991; Shishliaeva-Matova et al. 1966; Strelkova et al. 2015; Zhirenkina et al. 2011). These diseases are caused by different and usually not overlapping sets of *Leishmania* spp. in the region: *L. infantum* complex for VL and *L. major* and *L. tropica* for CL serving as the etiologic agents for zoonotic (ZCL) and anthroponotic (ACL) leishmaniasis, respectively (Akilov et al. 2007; Bruschi and Gradoni 2018; Ghatee et al. 2020; Yurchenko et al. 2023).

The geographical focus of this work is Surxondaryo (Surkhandarya) Region of Uzbekistan. This choice was determined by the following factors: (1) the highest prevalence and alarmingly positive dynamics of leishmaniasis here compared to other regions (Suvonkulov et al. 2020) and (2) the availability of historical studies for comparative analysis with a drawback of many papers published in Russian and never translated into English (Kogay 1960; Latyshev and Krukova 1941; Lugina 1959). As a matter of fact, the seminal discovery that two different subspecies (now, species) of *Leishmania*—*L. tropica minor* (now, *L. tropica*) and *L. tropica major* (now, *L. major*)—cause ACL and ZCL was done in Termez (now, Termiz; low Surxondaryo Region) by Yakimov and Schokhor, 1914 (Yakimov and Schokhor 1914). The systematic records of disease prevalence in this recognized focus of ZCL date back to 1932 (Dukelski and Rait 1932; Latyshev and Krukova 1941; Nasyrov and Yusupov 1974). Before the implementation of the mandatory prophylactic measures in the USSR in 1960–1970s (Kellina and Morozov 1982; Sergiev et al. 2018), the ZCL in Surxondaryo Region was not as widespread as in other regions of the country and restricted to just six reported localities (Ipatov and Zviagintseva 1967; Isaev 1959). It should be noted though that this number is most likely a severe underestimate due to the lack of reporting in many areas. The disease was practically eradicated by the mid-1980s (Sergiev et al. 2018; Sharipov et al. 1986), yet, following the collapse of the Soviet Union, it has returned “with vengeance” (Mustanov and Nematov 2019; Suvonkulov et al. 2020).

One of the potential reasons for such a comeback is the population dynamics of *Leishmania* spp. vectors. Indeed, complex measures targeting both the vectors and animal reservoirs applied in the second half of the last century led to a sharp drop in leishmaniasis prevalence (Sharipov et al. 1986). In this work, we investigated the phlebotomine sand fly (Diptera: Phlebotominae) diversity in the foci of cutaneous leishmaniasis in the Surxondaryo Region of Uzbekistan and compared it with the data obtained for the same area 50 years ago, when infection prevalence was reportedly low. We believe that these data will be valuable for developing effective control measures for sand fly in their natural habitats.

## Methods

**Fig. 1:**
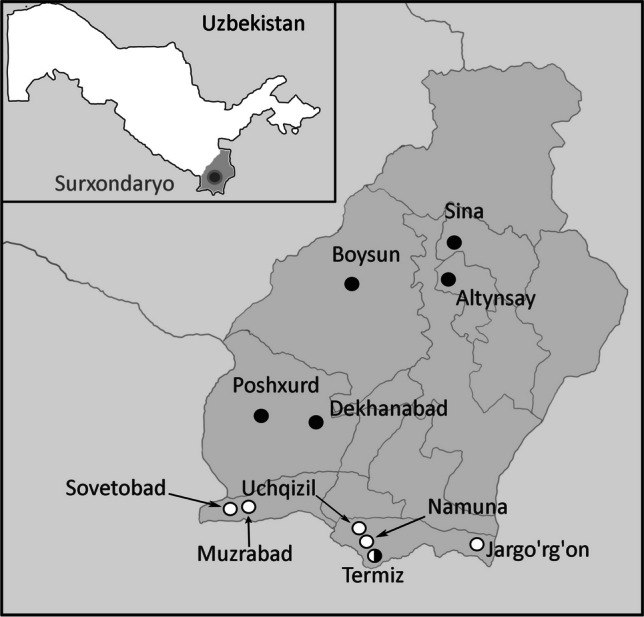
Map of collection localities in Surxondaryo Region in 1966-1972 (white dots) and 2021-2022 (black dots). Surxondaryo Region is the southmost part of Uzbekistan (insert)

The Surxondaryo is southernmost Region of Uzbekistan (Fig. 1). It features diverse landscapes from the plains of the Amu Darya river valley in the central and southern parts to the mountain ranges in the northern, eastern, and western parts. The climate here is warm with temperatures reaching + 45–60 °C in July; winters are short. In the plains and mountain foothill areas, the precipitation ranges from 130–360 mm to 440–620 mm annually, respectively.

Sand flies were collected in the summer months (June–August) of 2021 and 2022 in six stationary points in the Surxondaryo Region (Altynsay [38.2390° N, 67.6055° E, Altynsay District]—1 household; Boysun [38.1977° N, 67.2014° E, Boysun District]—1 household; Dekhanabad [37.6785° N, 67.0536° E, Sherabod District]—2 households; Poshxurd [37.7002° N, 66.7770° E, Sherobod District]—2 households; Sina [38.3603° N, 67.6841° E, Denov District]—3 households; Termiz [37.2611° N, 67.3086° E, Termiz District]—2 households), where leishmaniasis cases were registered (Fig. 1). The households, in which sand flies were caught, are traditional for Uzbekistan and consist of a residential building, sheds for keeping large and small livestock, a poultry house, a shed for firewood storage, and an outdoor toilet. There are usually several fruit trees and a vegetable/ornamental garden in the yard. Pets (typically, cats and dogs) are kept outside. The abundance of organic matter in the places protected from the direct sunlight and high humidity creates optimal conditions for the development of sand flies. Sand fly collection, fixation in gum arabic, and species identification were performed as described previously (Artemiev and Neronov 1984; Zvancov 2019). In short, sticky traps (approximately, 300 in total) were placed at a height of 50 to 150 cm in the evening and collected the following morning. Trapped sand flies were fixed in ethanol first before permanent fixation in the Fora-Berleze gum Arabic medium. Potential sand fly breeding sites (premises for keeping animals, sheds (including those used for dung storage), toilets, and residential areas) were examined separately. Historical data from 1966 to 1971 (Dzhabarov 1972; Zviagintseva 1968) (based on the analysis of 13,500 sand fly specimens in total) are shown in Table 1.

**Table 1:**
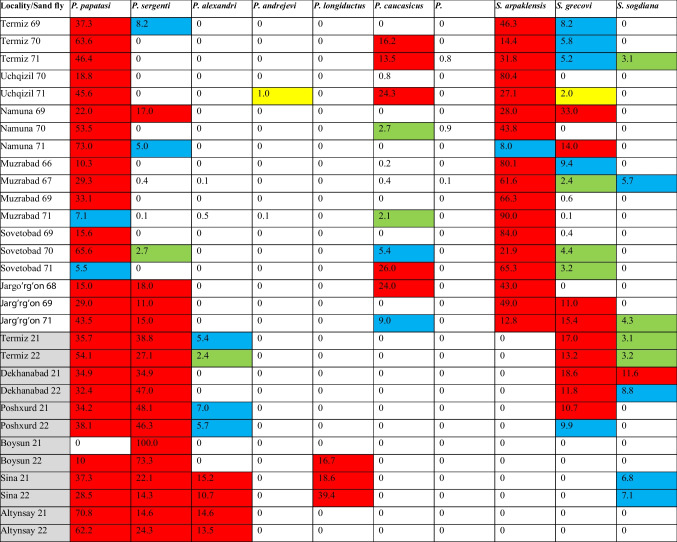
Species composition and relative abundance (in percent) of sand flies collected in the Surxondaryo Region. Collection sites and years of collection are indicated in the first column: 66, 1966; 67, 1967; 68, 1968; 69, 1969; 70, 1970; 71, 1971; 72, 1972; 21, 2021; 22, 2022 (grey background). Dominance of sand fly species in color-coded: eudominant (over 10%) are in red; dominant (5–9.9%) are in blue; subdominant (2–4.9%) are in green; recedent (1–1.9%) are in yellow; subrecedent (below 1%) are on the white background

## Results and discussion

### Analysis of sand fly diversity in the Surxondaryo Region of Uzbekistan in 2021–2022

A total of 969 sand flies of the two genera *Phlebotomus* (4 spp.) and *Sergentomyia* (2 spp.) were collected and analyzed (Table 1). Expectedly, two *Phlebotomus* sp.: *P.* (*Phlebotomus*) *papatasi* and *P.* (*Paraphlebotomus*) *sergenti* constitute the vast majority (over 80%) of the collected material since it was focused on the households with reported cases of leishmaniasis. This is a good representation of the 11 species of sand flies (*P.* (*Paraphlebotomus*) *alexandri*, *P.* (*Paraphlebotomus*) *andrejevi*, *P.* (*Paraphlebotomus*) *cauacasicus*, *P.* (*Paraphlebotomus*) *mongolensis*, *P.* (*Phlebotomus*) *papatasi*, *P.* (*Paraphlebotomus*) *sergenti*, *P.* (*Larroussius*) *smirnovi*, *S.* (*Sergentomyia*) *arpaklensis*, *S.* (*Sintonius*) *clydei*, *S. grecovi*, and *S. sogdiana*) previously documented in the Uzbek CL foci (Shishliaeva-Matova et al. 1966; Zviagintseva 1968). Five species not detected in our dataset always represented minor fractions in the collections analyzed earlier. The basic criteria for defining the vector include proof that the geographical distributions of the vector and the human disease overlap, that the sand fly species is anthropophilic, and that the same *Leishmania* sp. has been repeatedly isolated and identified in sand flies and patients. Supporting observations should demonstrate that the specific sand fly commonly feeds on reservoir hosts and can support both the development and transmission of the parasite (Killick-Kendrick 1990; Ready 2013). Sand flies can display a strong preference for a given *Leishmania* sp. (specific vectors) or allow the development of several *Leishmania* spp. (permissive vectors) (Alexandre et al. 2020; Dobson et al. 2010; Dostálová and Volf 2012; Dvorák et al. 2018; Kamhawi et al. 2000; Volf and Myšková 2007). Proven vectors of *L. major* and *L. tropica* in Central Asia are *P. papatasi* and *P. sergenti*, respectively (Maroli et al. 2013). In addition, three species of the *P. caucasicus* complex (*P. caucasicus*, *P. mongolensis*, and *P. andrejevi*) were shown to transmit *L. major* to animal hosts, but their involvement in the transmission to humans has not been demonstrated (Dvorák et al. 2018; Strelkova 1996), although *P. caucasicus* and *P.* (*Ph.*) *salehi* are considered proven vectors of *L. major* in Iran (Maroli et al. 2013). The proven vectors of *L. infantum* among the Asian species are *P*. *alexandri*, *P.* (*Adlerius*) *chinensis*, *P.* (*Ad.*) *sichuanensis*, *P.* (*La.*) *smirnovi*, and *P.* (*La.*) *wui* in China; *P.* (*Ad.*) *longiductus* in Kazakhstan; *P.* (*La.*) *major* s.l. and *P.* (*La.*) *transcaucasicus* in Iran; and *P.* (*Ad.*) *turanicus* in Turkmenistan (Maroli et al. 2013). *Phlebotomus* (*Ad.*) *longiductus* is the vector of *L*. *infantum* in Kazakhstan and western China (Dergacheva and Strelkova 1985; Guan et al. 2016) and *L. donovani* in Himalayan regions of India (Sharma and Singh 2008). This species is also considered the most likely vector transmitting VL in the Surxondaryo Region (Dergacheva and Strelkova 1985; Maroli et al. 2001). Of note, the competence of *Leishmania* spp. vectors is not fixed and may vary in different ecological and epidemiological settings (Ready 2013).

Most of the collected sand flies were males (ranging from 61% for *P. papatasi* to over 90% for *S. grecovi*) (Suppl. Table 1). This confirms previous report from this and other areas on difference in mobility of sand fly sexes (Boussaa et al. 2016; Maroli et al. 2001; Yared et al. 2017). Sand fly distribution in different parts of the household (Suppl. Table 1) is also unremarkable. Except for *P. longiductus* (absent in living areas) and *S. sogdiana* (caught only in sheds and yards), all other species were most abundant in the sheds. However, the low number of collected specimens precluded us from making solid conclusions in this regard.

It should be noted that most of the previous studies were focused on southern (low) Surxondaryo Region (Dzhabarov 1972; Ipatov and Zviagintseva 1967; Isaev 1959; Zviagintseva 1968). The presented study extended the analyzed area northward. This is where (Boysun and Sina) a likely vector for VL-causing *L. infantum*, *P. longiductus*, was found. Notably, its prevalence is comparable to that documented in the VL foci of Pap district (Fergana Valley) of Namangan Region, Uzbekistan (Maroli et al. 2001). In addition, the fraction of *P. sergenti* (associated with *L. tropica* and ACL) in Boysun was shown to be extremely high (over 87%) indicating an active focus of ACL in this locality.

### Analysis of sand fly diversity in the Surxondaryo Region of Uzbekistan: comparative study

In this study, our primary question focused on whether the increase in leishmaniasis cases in the Surxondaryo Region is linked to the population dynamics of *Leishmania* vectors. In comparison with 1966–1971 studies (Dzhabarov 1972; Zviagintseva 1968), we noted the following: (i) the implicated ZCL vector, *P. papatasi*, remained eudominant (with a sole exception of Boysun), where it was shown to be subdominant, but this locality has not been studied previously; (ii) the proportion of implicated ACL vector, *P. sergenti* (now, eudominant in all the sampled locations), rose significantly from averaged 5.4% (Dzhabarov 1972) to 41.4%; (iii) neither *P. andrejevi* nor *P. caucasicus*, *P. mongolensis*, or *S. arpaklensis* were detected in 2021–2022; (iv) the proportion of two *Sergentomyia* spp., *S. grecovi* and *S. sogdiana*, implicated in transmission of reptile parasites of the subgenus *Leishmania* (*Sauroleishmania*) (Maroli et al. 1988; Ovezmukhammedov and Saf’janova 1989; Tichá et al. 2023) became more prominent while *S. arpaklensis* has not been found even in Termiz, where it was previously eudominant; (v) *Phlebotomus alexandri*, a suspected VL vector, was eudominant at two sites and a second suspected vector for the VL-causing *L. infantum*, *P. longiductus*, was newly recorded in the region.

We conclude that the increase in the documented cases of leishmaniasis in the Surxondaryo Region of Uzbekistan may be connected to the changes in the fauna of sand flies vectoring *Leishmania* spp. although the reasons underlying this must be investigated further. It implies that more drastic measures of control and detection (in humans, animal reservoirs, and vectors) should be implemented to slow down or stop this alarming trend. The fight against leishmaniasis is a complicated challenge. There are still no vaccines available and the transmission of the disease is a complex chain of many elements. From an epidemiological and public health point of view, it is important to establish new prevention and control programs. People should use mosquito nets to protect dwellings and repellents for outdoor activities at dusk when vector activity is high.

### Supplementary Information

Below is the link to the electronic supplementary material.

Supplementary file1 Suppl Table 1. Sand flies’ habitats. The numbers of caught sandflies are shown for 1 – living area; 2 – shed; 3 – poultry house; 4 – firewood storage; 5 – yard; 6 – toilet. The proportion of collected male sand flies is shown in parentheses in the second column. (DOCX 22 KB)

## Data Availability

The data supporting the findings of this study are available within the article and its supplementary materials.
