# Supplementary material for: Phlebotomine sand fly (Diptera: Phlebotominae) diversity in the foci of cutaneous leishmaniasis in the Surxondaryo Region of Uzbekistan: 50 years on
Source: Parasitol Res. 2024 Mar 25;123(3):170. doi: 10.1007/s00436-024-08191-4 (PMC10963468; doi:10.1007/s00436-024-08191-4)
Supplement: Supplementary file 1 — Supplementary file1 Suppl Table 1. Sand flies’ habitats. The numbers of caught sandflies are shown for 1 – living area; 2 – shed; 3 – poultry house; 4 – firewood storage; 5 – yard; 6 – toilet. The proportion of collected male sand flies is shown in parentheses in the second column. (DOCX 22 KB) [file 436_2024_8191_MOESM1_ESM.docx]

| **Sand fly sp.** | **Total (% males)** | **1** | **2** | **3** | **4** | **5** | **6** |
| --- | --- | --- | --- | --- | --- | --- | --- |
| ***P. alexandri*** | 59 (81) | 4 (6.7) | 30 (50.9) | 8 (13.5) | 5 (8.5) | 12 (20.4) | 0 |
| ***P. longiductus*** | 16 (69) | 0 | 3 (18.7) | 3 (18.7) | 2 (12.5) | 5 (31.4) | 3 (18.7) |
| ***P.papatasi*** | 378 (61) | 32 (8.5) | 179 (47.3) | 46 (12.1) | 67 (17.8) | 31 (8.2) | 23 (6.1) |
| ***P. sergenti*** | 403 (78) | 31 (7.6) | 160 (39.7) | 78 (19.4) | 23 (5.7) | 55 (13.7) | 56 (13.9) |
| ***S. grecovi*** | 95 (90) | 6 (6.4) | 46 (48.5) | 9 (9.6) | 16 (16.3) | 9 (9.6) | 9 (9.6) |
| ***S. sogdiana*** | 18 (83) | 0 | 9 (50.0) | 0 | 0 | 9 (50) | 0 |
| **Total** | **969** | **73** | **427** | **144** | **113** | **121** | **91** |
